# Reaching higher: External scapula assistance can improve upper limb function in humans with irreversible scapula alata

**DOI:** 10.1186/s12984-021-00926-z

**Published:** 2021-09-03

**Authors:** Anna-Maria Georgarakis, Michele Xiloyannis, Christian Dettmers, Michael Joebges, Peter Wolf, Robert Riener

**Affiliations:** 1 Sensory-Motor Systems (SMS) Lab, Institute of Robotics and Intelligent Systems (IRIS), Department of Health Sciences and Technology (D-HEST), ETH Zurich, Zurich, Switzerland; 2 Reharobotics Group, Spinal Cord Injury Center, Balgrist University Hospital, Medical Faculty, University of Zurich, Zurich, Switzerland; 3 Kliniken Schmieder Konstanz, Konstanz, Germany

**Keywords:** Muscular dystrophy, Muscle weakness, Scapula alata, Winging scapula, Scapula orthosis, Scapula Assistance Test, Scapular fusion, Daily life

## Abstract

**Background:**

Scapular dyskinesis, i.e., the deviant mobility or function of the scapula, hampers upper limb function in daily life. A typical sign of scapular dyskinesis is a scapula alata—a protrusion of the shoulder blade during arm elevation. While some reversible causes of scapula alata can be treated with therapy, other, irreversible causes require invasive surgical interventions. When surgery is not an option, however, severe limitations arise as standard approaches for assisting the scapula in daily life do not exist. The aim of this study was to quantify functional improvements when external, i.e., non-invasive, scapula assistance is provided.

**Methods:**

The study was designed as a randomized controlled crossover trial. Eight participants with a scapula alata due to muscular dystrophy performed arm elevations in shoulder flexion and abduction while unassisted (baseline), externally assisted by a trained therapist, and externally assisted by a novel, textile-based scapula orthosis.

**Results:**

With therapist assistance, average arm elevation increased by 17.3**°** in flexion (p < 0.001, 95% confidence interval of the mean $C{I}_{95\%}=\hspace{0.17em}\left[9.8^\circ , 24.9^\circ \right]$), and by 11.2° in abduction (p < 0.01, $C{I}_{95\%}=\left[4.7^\circ , 17.7^\circ \right]$), constituting the potential of external scapula assistance. With orthosis assistance, average arm elevation increased by 6.2° in flexion ($C{I}_{95\%}=\left[0.4^\circ ,11.9^\circ \right]$) and by 5.8° in abduction ($C{I}_{95\%}=\left[3.0^\circ ,8.5^\circ \right]$). Remarkably, in three participants, the orthosis was at least as effective as the therapist. Moreover, orthosis assistance reduced average perceived exertion by 1.25 points (Borg Scale) when elevating a filled bottle during a simulated daily living task.

**Conclusion:**

These findings indicate a large potential for future advancements in orthotics. Already now, the textile-based scapula orthosis presented here is a feasible tool for leveraging the benefits of external scapula assistance when a therapist is unavailable, as encountered in daily life scenarios.

*Trial Registration* ClincalTrials.gov (ID NCT04154098). Registered: November 6th 2019, https://clinicaltrials.gov/ct2/show/NCT04154098?term=scapula+orthosis&draw=2&rank=1

**Graphic abstract:**

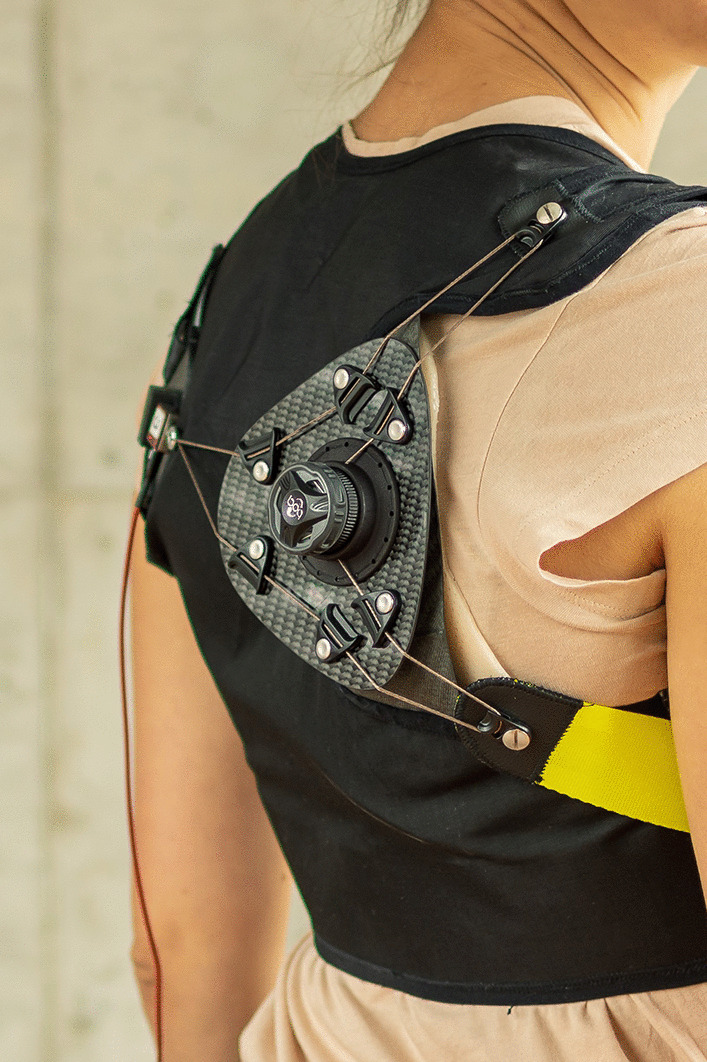

**Supplementary Information:**

The online version contains supplementary material available at 10.1186/s12984-021-00926-z.

## Background

The dynamic stability of the shoulder joint complex is the bedrock for the versatile mobility of the human upper limb [1]. Without stability, confident, well-controlled arm movements are not achievable. Consequently, humans with scapular dyskinesis, i.e., an altered scapular position or scapular dynamic motion [2], are hampered during daily, recreational, and occupational activities, ultimately limiting their quality of life.

The mechanics of the upper limb can essentially be described as an open kinematic chain that is attached to the trunk solely at the sternum [3]. Connected to the trunk via the clavicle, the upper arm rotates within the scapula, a conjunction that can be approximated as a ball and socket joint. This inherently unstable structure is primarily kept together by the co-contracting muscles of the upper trunk and shoulder [1].

In this complex, the scapula plays a key role [4]. It serves as the floating base for the humerus, the upper arm bone. During arm elevation, the scapula rotation contributes to about a third of the total humeral elevation. This movement pattern is called the scapulohumeral rhythm [1, 5, 6]. When the coordination of the scapulohumeral rhythm is disrupted, for example due to a muscular or neurological disease, the function of the entire upper limb is fundamentally impaired. Often, these functional limitations are countered with compensatory trunk movements [3, 7].

A typical symptom of a disrupted scapulohumeral rhythm is a scapula alata, or winging scapula [8, 9]. A scapula alata is caused by a dysfunctional activation of the muscles that coordinate the scapular movement on the thoracic wall. The underlying impairment can be of muscular, neuronal, or coordinate origin. A scapula alata is characterized by and excessive posterior tilt of the scapula during forward arm flexion, or an excessive scapular medial rotation during sideward arm abduction. For example, an isolated, unilateral scapula alata is the hallmark of a long thoracic nerve palsy with secondary paresis of the serratus anterior muscle. A bilateral, symmetrical, slowly progressive scapula alata may be observed in myopathies with proximal manifestation, in particular in facioscapulohumeral dystrophy (FSHD).

When the scapula alata is reversible at least in part, for instance in stroke, in traumatic nerve palsy or in a spontaneous motor control deficit [10, 11], physical therapy can help to relearn physiological movement patterns. During this process, physical therapy can be effectively complemented by functional electrical stimulation [12] or by wearing a scapula brace [13–17].

When the scapula alata is irreversible, for instance in muscular dystrophy or an irreversible nerve damage [18], physical therapy aims at preserving arm function by stretching, preventing excessive malfunction and reducing pain, and at learning compensatory movements to increase upper limb function. Compensatory movements, however, are energy-inefficient and often come at the cost of muscle tensions and excessive joint loads [4].

To treat an irreversible scapula alata, several solutions have been proposed to constrain the scapula against the thoracic wall. Similarly to therapy methods in reversible winging, in irreversible winging, the trunk can be braced to apply a pressure above the scapula [9]. However, to provide the forces required to prevent scapular winging, typical braces consist of large rigid structures, which are not always well tolerated by patients [14]. The most prominent treatment of an irreversible scapula alata is surgery, in which muscles and ligaments are re-routed to constrain the winging of the scapula during arm elevation [19], or in which the scapula is fused directly with the thoracic wall [20–27]. Though most reports on scapular fusion have very positive outcomes, they were acquired from observational studies on highly selected patient groups. To date, randomized controlled trials providing evidence on the outcomes of surgical interventions are unavailable [18]. Given their very invasive nature, surgical interventions are only considered in extreme cases of immobility or pain.

So far, it is not well understood to what extend less invasive, external scapula assistance can increase upper limb function in humans with an irreversible scapula alata. Here, external scapula assistance refers to a fully reversible, non-invasive, extracorporeal form of instantaneous support for the scapula. To shed light on the unknown, this randomized crossover study aimed to compare the movement capabilities of participants with an irreversible scapula alata when unassisted, when externally assisted by a trained therapist (therapist assistance), and when externally assisted by a fully mobile, wearable orthotic device (orthosis assistance). We hypothesized that with dynamic therapist assistance, i.e., by manually assisting the scapula on the physiological movement path, participants with an irreversible scapula alata could increase their range of motion in shoulder elevation. With orthosis assistance, i.e., by statically constraining the scapula on the thoracic wall, range of motion was expected to increase, but to a lesser extent than with therapist assistance. Since stabilizing the scapula affects the whole kinematic chain of the arm, it was additionally hypothesized that both forms of external scapula assistance, i.e., therapist and orthosis assistance, reduce trunk compensation and movement effort, and improve movement smoothness. These functional improvements become particularly apparent when arm elevations are performed under load [28, 29].

By investigating the effect of external scapula assistance on humans with an irreversible scapula alata, a general understanding for the potential of static, i.e., solely constraining the scapula on the thoracic wall, and dynamic, i.e., causing a rotation of the scapula on the thoracic wall, scapula assistance can be established. The collected evidence may launch the comprehensive development and enhancement of devices for permanent scapula assistance in daily life, whose applicability may extend to the rehabilitation of reversible scapula conditions and to injury prevention during vocational lifting tasks.

## Methods

### Study design

**Fig. 1:**
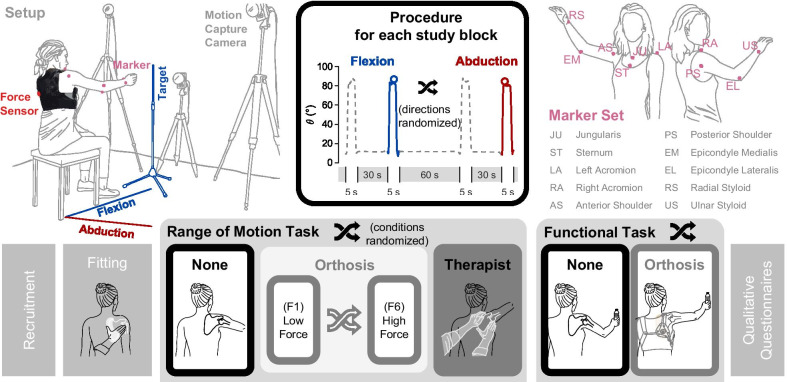
Study protocol and setup. Participants were recruited through the clinical partner. The orthosis was customized by fitting the scapular interface to each participant, see also Fig. 2. The range of motion task consisted of one study block without assistance, six study blocks with orthosis assistance set to different force levels, and one study block with therapist assistance. During each study block, participants performed two maximal arm elevations in both the flexion and abduction direction. The functional task consisted of one block without assistance and one block with orthosis assistance set to the force level rated most helpful by the participant. During each study block, participants performed two maximal arm elevations in flexion direction while holding a weighted bottle. Additionally, participants completed a qualitative questionnaire at the end of the study. Participants’ movements were recorded with a six-camera motion capture system and a ten-marker set

**Fig. 2:**
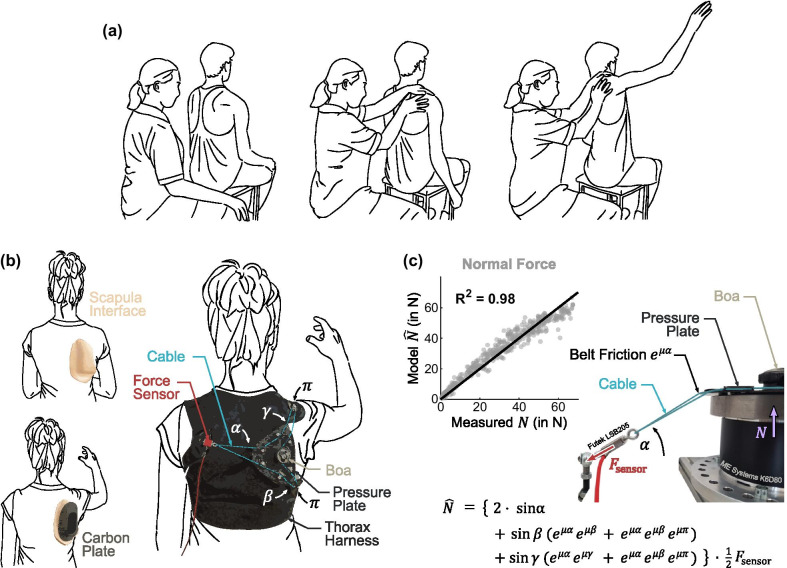
Scapula assistance methods. **a** Therapist assistance: To dynamically stabilize the scapula, the therapist performed the modified Scapular Assistance Test (mSAT). Using the left hand as counteraction, the therapist applied an assistive force on the medial border and inferior angle of the scapula with the right hand, therefore constraining and rotating the scapula on the thoracic wall. **b** Scapula orthosis design: Similar to the therapist’s hand, the customized scapula interface girted the scapula’s medial border and inferior angle. A carbon plate was used to reinforce the scapula interface to allow for better force distribution. The top layer comprised a thorax harness in which a cable-based Boa-Fastening mechanism, mounted on a carbon pressure plate, was threaded. **c** Force model validation: With information from the force sensor and the geometry of the pressure mechanism, the applied normal force was calculated

The study was designed as a randomized controlled crossover trial. Two study tasks were defined, see Fig. 1. In the range of motion task, participants performed one block of arm elevations without assistance (“None” condition), a set of six blocks with scapula orthosis assistance (“Orthosis” condition), and one block with therapist assistance (“Therapist” condition). The wearable, textile-based scapula orthosis, see Fig. 2b, was continuously adjustable in size and force level. While a low force level may not suffice to constrain the winging scapula sufficiently, a high force level may result in exuberant tension on the thorax harness, therefore restricting the participants movement. Hence, to determine the effect of force application on upper limb function, for each participant, six different orthosis force settings were tested. In the functional task, participants elevated their arm while holding a filled bottle. To take their level of ability into consideration, participants could pick between a half-full (mass ${m}_{\mathrm{half}}=0.25 \mathrm{kg}$) and a full (${m}_{full}=0.50 \mathrm{kg}$) bottle. Participants performed one block of arm elevations without assistance and one block with the orthosis set to the force level rated most helpful by the participant. For each task, conditions were randomized, and the set of orthosis blocks was randomized within.

During each of the eight blocks in the range of motion task, participants performed two maximal arm elevations for 5 s in both flexion and abduction in randomized order. Due to the tiring nature of the functional task, only the flexion direction was presented there. Between arm elevations, participants rested for 30 s, with a longer break of 60 s when the target direction was swapped.

Before the experiment, participants provided demographic information as well as records of their disease progression. Before the first study block, the orthosis interface was customized for each participant, the textile thorax harness, see Fig. 2b, was adjusted to the participant to the experimenter’s best knowledge and ability, and participants were verbally instructed about the study procedure. After each study block, participants completed the Nordic Questionnaire [30] and the Borg Scale [31]. After the last study block, participants completed a qualitative questionnaire about the scapula orthosis.

### Study setup

Participants were seated on a stool without backrest. Following the vertical projection of the participants’ glenohumeral joint, the flexion and abduction directions were marked on the floor with tape. A target pole, placed at the end point of the current target direction, accentuated the targeted direction of arm elevation. Six motion capture cameras (Oqus 300, Qualisys, Sweden), set up around the participants’ right arm, recorded the movement at 100 Hz. As both the clavicle and scapula were covered by the therapist and orthosis assistance, only arm motion was captured [32, 33]. Due to the morphological changes that occur with the progression of a muscular dystrophy, and to avoid premature fatigue of the participants, instead of calibrating the marker positions with calibration movements, ten anatomical landmarks were chosen as reference locations for the markers (Super spherical markers, Ø 14 mm, Qualisys, Sweden), see Fig. 1 [33].

### Study in- and exclusion criteria

Inclusion criteria included the following: at least 18 years of age, diagnosed scapula alata, ability to sit on a stool without back rest for at least two hours, limited range of motion in at least one of the upper extremities, passive shoulder elevation range of at least 110°. Exclusion criteria included a frozen shoulder, osteoporosis and arthrosis of the shoulder joint, shoulder subluxation, skin ulcerations on the affected arm or torso and pregnancy. The study was approved by the ETH Zurich ethics commission (EK 2019-N-11) and pre-registered on ClincalTrials.gov (ID NCT04154098). Participants provided written, informed consent prior to the experiments.

### Requirements for external scapula assistance

In their biomechanical analysis, Barnett et al. found that a force of 100 N in anterior direction and a force of 82 N in lateral direction must be applied at the inferior angle of the scapula to counteract the torques that emerge during arm elevation [9]. It is assumed that patients with muscular dystrophy are still able to produce a share of the required force to restrain the scapula to the thoracic wall. Hence, the forces found by Barnett represent an upper bound for the forces required to externally stabilize the scapula. Furthermore, to apply an external force that prevents medial or dorsal winging, a counterforce must be applied whose resultant is acting in the opposite (lateral or ventral) direction.

To minimize the risk of skin ischemia and blood occlusion, short term pressures on the skin should not exceed 16 kPa [34], while long term pressures should not exceed 4 kPa [35]. Due to the focus of force application, the area above the scapula is subject to the largest occurring pressures in the orthosis. The scapular interface used to transfer the restraining forces should therefore be large enough to distribute the forces such that any occurring pressure remain within these bounds. Furthermore, the scapula interface should conform to the scapular curvatures to distribute pressures and avoid pressure peaks. Moreover, it is desirable to enable adjustment in the force application to increase comfort and user compliance [9].

### Therapist assistance

**Fig. 3:**
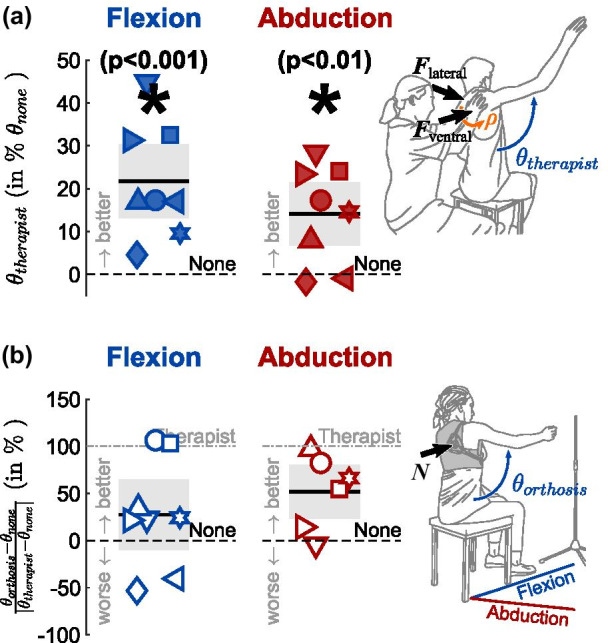
Effect of external scapula assistance. **a** Normalized effect of therapist assistance: Improvement from baseline with therapist assistance, normalized by baseline ability. The assistive forces applied by the therapist in ventral ${{\varvec{F}}}_{ventral}$ and lateral ${{\varvec{F}}}_{lateral}$ direction provided dynamic scapula rotation $\rho$ while the participant elevated their arm by ${\theta }_{therapist}$. **b** Relative effect of orthosis assistance: Improvement from baseline with orthosis assistance, normalized by maximal therapist improvement. The assisitve normal force ${\varvec{N}}$ applied on the scapula interface constrained the scapular winging while the participant elevated their arm by ${\theta }_{orthosis}.$ In **b**, data with negative therapist improvement in **a** were not considered to prevent discrepancies in data interpretation. **a** and **b** Black bar: mean; grey boxes: 95% confidence interval of the mean

During the range of motion task, the therapist bimanually assisted scapular rotation while the participants elevated their arm in flexion and abduction direction. To this end, the therapist performed a modified Scapular Assistance Test (mSAT), see Fig. 2a. The mSAT was developed to diagnose scapula dyskinesia by assessing shoulder pain during arm elevation when a gentle force is applied [4, 10, 28]. Here, the therapist applied a larger guiding force to assist scapular rotation. While applying a counterforce with the left hand, the therapist guided the scapula during lateral rotation in abduction and during lateral and ventral rotation around the thorax in flexion by applying a pressure on the medial border and inferior angle of the scapula with the right hand, see Fig. 3a. As in the orthosis condition, the elevation of the arm itself was performed autonomously by the participant.

### Scapula orthosis design

In discussion with patients, clinicians and orthopedic technicians, additional practical requirements for the design of the orthosis were revealed. The orthosis should be made of a flexible material that allows for adjustments to different body types. Rigid parts should be reduced to a minimum and conform with the user’s body shape. To facilitate breathing, any tight construction around the thorax should be avoided or released when the orthosis is not in use. Sensitive regions such as the breast area should be spared.

Based on the requirements, the orthosis was designed in three layers. The first layer comprised a rigid scapula interface. The scapula interface was manufactured from orthopedic thermoplastic material (micro-perforated, 2 mm, Orfit, Belgium) that was directly fitted to the participant’s scapula and reinforced with a carbon plate, see Fig. 2b. Using a rectangular molding aid, the medial border of the scapula was girted. This way, applying a pressure onto the posterior face of the scapula interface resulted in constraining forces that counteracted both medial and dorsal winging.

The second layer comprised a textile thorax harness that anchored the counterforces acting on the anterior and lateral side of the body. The harness was manufactured from compliant, non-extensible material (Cordura 500den, Invista, Switzerland) with adjustable straps around the shoulders and chest. Sewing patterns for the harness can be found in Additional file 1.

The third layer comprised a rigid pressure plate with a rotational cable-fastening mechanism (H3, Boa, USA). The cable (Snag Leader, Zeck Fishing GmbH, Germany) was routed between the pressure plate and three anchor points on the thorax harness, see Fig. 2b, thus constituting a continuously adjustable, force-enhancing pulley system.

### Force measurement mechanism and normal force model

Since the pressure plate was elevated with respect to the harness anchor points, the resultant force on the pressure plate comprised an effective normal force component ${\varvec{N}}$ acting on the scapula interface, see Fig. 3b. A normal force model was used to relate the effective normal force to the force measured on the cable in the pulley system, see Fig. 2c. The pulley system was modeled taking the system geometry and belt friction into account. The angles $\alpha , \beta \mathrm{and} \gamma$ between the thorax anchor points and the pressure plate were measured for each ‘Orthosis’ study block. The friction coefficient for the cable was set to $\mu =0.1$ [36].

To validate the normal force model, the pressure plate was mounted on a 6-axis force-torque sensor (K6D80, ME systems, Germany). Using a 1-axis force sensor (LSB205 FSH04422, Futek, USA) to measure the tension on the cable, the normal force model showed good validity over the assessed force range (${R}^{2}=0.98$), see Fig. 2c.

### Angle definitions

The arm elevation angle with respect to gravity ${\theta }_{\mathrm{g}}$ was defined locally in the plane of elevation as the angle between the wrist vector, connecting the shoulder and the wrist, and the gravity vector during peak arm elevation. The plane of elevation was defined as the plane spanned by the wrist vector and the gravity vector. The wrist vector was chosen for several reasons. Firstly, the wrist elevation is a more functional outcome than the elevation of the upper arm (humerus). Secondly, the wrist plane of elevation deviated less (deviation mean and standard error: − 8.7°|3.2° for flexion, 6.8°|3.1° for abduction) from the target plane than the humeral plane of elevation (− 27.5°|4.0° for flexion, − 5.5°|4.3° for abduction) when participants elevated their arm in the direction of the target pole, see Additional file 3. Lastly, and most importantly, the arm was considered a kinematic chain, hence a manipulation of the proximal shoulder joint likewise influences the more distal joints at the elbow and wrist.

The trunk compensation angle $\kappa$ was defined as the differential of the angle between the sternum and the gravity vector between current arm elevation and the initial position, projected onto the plane of elevation. The arm elevation angle $\theta$ was defined as

$$\theta = \theta_{g} - \kappa$$

that is the gravitational elevation angle ${\theta }_{\mathrm{g}}$ corrected for the thorax compensation angle $\kappa$. The scapula rotation angle $\rho$ was defined as the angle between the medial border of the scapula and the gravity vector. The glenohumeral elevation angle ${\theta }_{\mathrm{GH}}$ was defined as

$$\theta_{{{\text{GH}}}} = \theta_{{\text{g}}} - \rho$$

which corresponds to the angle between the humerus and the scapula rotation angle $\rho$, see also Table 1.

**Table 1:** Study participant characteristics, pathology and ability

	Characteristics			Pathology		Ability		
	Sex	Height (m)	Weight (kg)	Diagnosis	Chronicity (years)	Brooke Scale	${\theta }_{GH}$:$\rho$^a^ (°:°)	
P1	m	1.83	74	FSHD	9	3	1:0.2	
P2	m	1.82	63	FSHD	21	3	1:− 0.4	
P3	f	1.58	53	MYOS	18	4	1:− 0.3	
P4	m	1.80	110	FSHD	10	3	1:− 0.2	
P5	f	1.71	49	CALP	13	3	–	
P6	f	1.68	60	FSHD	37	3	1:− 0.4	
P7	m	1.89	115	FSHD	27	3	1:− 0.3	
P8	f	1.75	73	FSHD	28	5	1:− 0.4	

*FSHD* facio-scapulo-humeral dystrophy; *MYOS* necrotizing myositis; *CALP* calpainopathy

^a^ScAla score

The plane of elevation angle $\alpha$ was defined as the angle between the projections of the line connecting both acromia in the initial position and the line connecting the glenoid with the wrist onto the horizontal plane. Flexion was defined in correspondence with the primary direction during arm elevation occurring in daily life at $\alpha =80^\circ$ plane of elevation angle [37]. Abduction was defined in correspondence to the scapular plane at $\alpha =30^\circ$ plane of elevation angle. An arm elevation in the scapular plane results in minimal strain and therefore movement resistance in the glenohumeral joint capsule [38]. A visualization of the angles defined in this study can be found in Additional file 2.

### Data analysis and statistics

Motion capture data was post-processed in QTM (v2019.3) and Matlab (R2020b). An additional analysis of motion capture errors can be found in Additional file 4. The position of the sternum, humerus and forearm were reconstructed from marker positions in accordance with ISB recommendations [39] to extract the sternum compensation angle $\kappa$ and the arm elevation angle ${\theta }_{\mathrm{g}}$ with respect to gravity.

For each study block, only the second elevation per target direction was included in the data analysis, see Fig. 1. After low-pass filtering the movement data at 5 Hz [7] and segmenting movements at the velocity threshold of 7°/s, elevation peaks were extracted directly.

For statistical testing, linear mixed effect models of the form

$$\vartheta \sim support*direction + \left( {1|participant} \right) + \in$$

were used, where $\vartheta$ is the response variable, $support$ is a fixed effect with the three levels “None”, “Therapist” and “Orthosis”, $direction$ is a fixed effect with the two levels flexion and abduction, $participant$ is a random intercept for each participant, and $\in$ is the residual error. Tests were performed using post-hoc hypothesis testing. The family-wise error rate was controlled using the Bonferroni–Holm method.

For the functional task, movement smoothness was determined utilizing the spectral arc length (SPARC) metric [40]. Movement data were segmented into arm ascent, hold and descent using a 10°/s threshold on the angular arm elevation velocity $\dot{\theta }$.

## Results

### Participant characteristics

Eight participants with muscular dystrophy were recruited through the clinical partner. All participants completed the study protocol. The adjustable orthosis was successfully fit to all participants despite their diverse morphologies, ranging from small females to large males (height 1.58…1.89 m, weight 49…115 kg), see Table 1. The orthosis was fit unilaterally to the right scapula for all participants, as in this study cohort, the right scapula was equally or more affected by winging than the left scapula. Participant’s ability was classified using the Brooke Scale [41]. Moreover, a Scapula Alata (ScAla) score was defined that quantifies the rotation of the scapula in relation to the angle of arm elevation, see the figure in Table 1. For an individual without impairment, the ScAla score is 1:0.5 in accordance with the scapulohumeral rhythm [1, 5, 6]. ScAla scores ranged from 1:0.2 to 1:− 0.4, where negative values indicate medial winging. For participant P5, the ScAla score could not be determined due to a lack of reference pictures.

### External scapula assistance improves range of motion in shoulder elevation

**Fig. 4:**
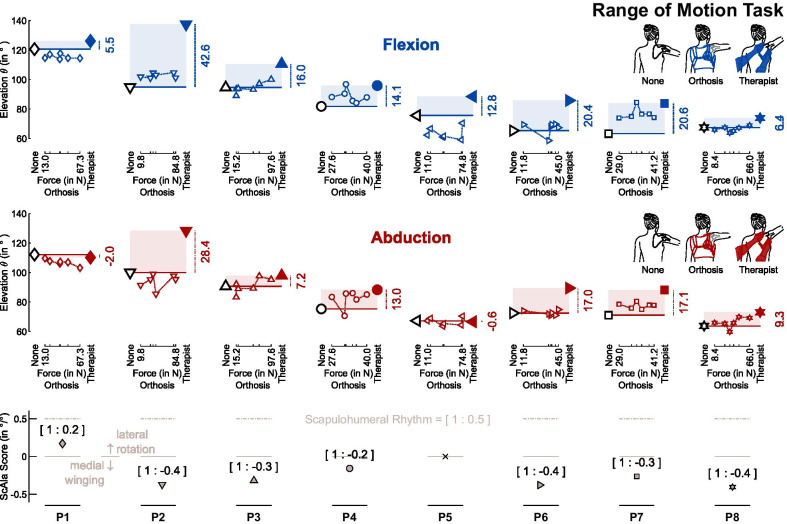
Range of motion results for baseline, Orthosis and Therapist assistance in flexion and abduction direction. Participants completed one block of arm elevation without assistance (black edge symbols), six blocks of arm elevation with orthosis assistance (colored-edge symbols) and one block with therapist assistance (filled symbols). Shaded boxes indicate the maximum improvement potential. The Scapula Alata (ScAla) score is illustrated in the bottom line for reference

When external scapula assistance was provided, the arm elevation $\theta$ increased both in arm flexion and abduction. With therapist assistance, the improvement to the baseline was significant both in flexion (p < 0.001, mean $\mu =17.3^\circ$, 95% confidence interval in terms of the standard error of the mean $C{I}_{95\%}=\left[9.8^\circ , 24.9^\circ \right]$) and in abduction direction (p < 0.01, $\mu =11.2^\circ$, $C{I}_{95\%}=\left[4.7^\circ , 17.7^\circ \right]$), see Fig. 3a. With orthosis assistance, improvements were lower than with therapist assistance in flexion ($\mu =6.2^\circ$, $C{I}_{95\%}=\left[0.4^\circ ,11.9^\circ \right]$) and abduction ($\mu =5.8^\circ$, $C{I}_{95\%}=\left[3.0^\circ ,8.5^\circ \right]$). Outcomes with orthosis assistance were more divergent, ranging from intra-participant mean improvements of up to 13.5°/6.8° (flexion/abduction), to intra-participant mean reductions of up to − 12.1°/− 6.0° when compared to the baseline without assistance. Therapist assistance was considered an upper bound for the effect of orthosis assistance. Intra-participant changes observed when receiving maximal orthosis assistance ranged from − 53.2% to 106.4% of those measured during therapist assistance in flexion, and from − 3.8% to 96.9% in abduction, see Fig. 3b. During the orthosis assistance condition, the orthosis was released before each block and a new force level was set upon re-tightening it, see Fig. 4. For the consequent intra-participant variability in peak arm elevation with orthosis assistance, the $C{I}_{95\%}$ width for participants ranged from 1.3° to 3.6° in flexion, and from 1.3° to 4.7° in abduction*.*

### External scapula assistance helps to reduce trunk compensation

To increase the workspace of their upper limb, patients with a scapula alata typically adopt compensatory trunk movements. The compensation serves to counterbalance the disruptive movement of the scapula. Therefore, compensatory movement patterns during arm elevation were expected to diminish when the scapula is stabilized externally.

Trunk compensatory movements were consistently reduced in three of the eight participants during arm elevation in flexion direction, and in all but one participant in abduction direction. Without assistance, compensation angles $\kappa$, extracted during peak arm elevation, ranged from 2.8 to 36.8° in flexion, and from 6.6 to 32.9° in abduction. With orthosis assistance, compensation angles $\kappa$ ranged from 0.6 to 28.7° in flexion, and from 1.3 to 29.8° in abduction. With therapist assistance, compensation angles $\kappa$ ranged from 5.1 to 21.3° in flexion, and from 6.0 to 28.5° in abduction.

**Fig. 5:**
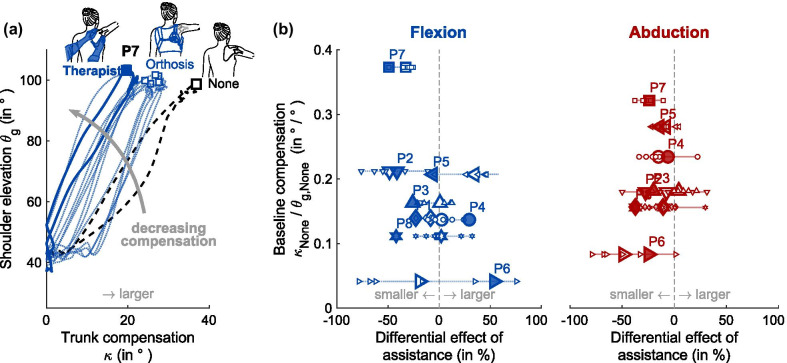
Trunk compensation decreased with external scapula assistance. **a** Trunk compensation can be described as a function of arm elevation. Exemplary data from participant P7. **b** To compare trunk compensation between different conditions, it was normalized by arm elevation. Dotted lines and white-faced markers represent changes in trunk compensation with orthosis assistance (small symbols: individual trials; large symbols: intra-participant means), while solid markers represent changes in trunk compensation with therapist assistance

As expected, trunk compensation typically increased with arm elevation, see Fig. 5a. To compare trunk compensation for different peak elevations, the trunk compensation angle $\kappa$ was normalized by the elevation angle ${\theta }_{g}$. Baseline compensation without assistance ranged from 0.04°/° to 0.37°/° in flexion, and from 0.08°/° to 0.32°/° in abduction. These values decreased both with orthosis (flexion 0.01°/° to 0.33°/°, abduction 0.02°/° to 0.29°/°) and therapist (flexion 0.06°/° to 0.19°/°, abduction 0.06°/° to 0.25°/°) assistance. On average, participants decreased their compensatory movements by 8.6% (flexion) and 15.1% (abduction) with orthosis assistance and by 13.0% (flexion) and 23.8% (abduction) with therapist assistance, see also Fig. 5b.

### External scapula assistance reduces perceived exertion in a functional task

The scapula constitutes a key element in the kinematic chain of the arm. Stabilization of the scapula during daily living tasks may thus not only influence the range of motion of the arm, but also movement effort. Therefore, after the range of motion task, participants completed another set of arm elevations while holding a bottle. Before the task, participants were able to choose the one out of two bottle weights they felt more confident to lift. Three participants picked the heavier bottle (500 g, P1, P7 and P5), the other five participants picked the lighter bottle (250 g). The functional task focused on elevation in flexion, as arm flexion is the most relevant movement direction for activities of daily living [37]. The task was performed both with and without orthosis assistance.

**Fig. 6:**
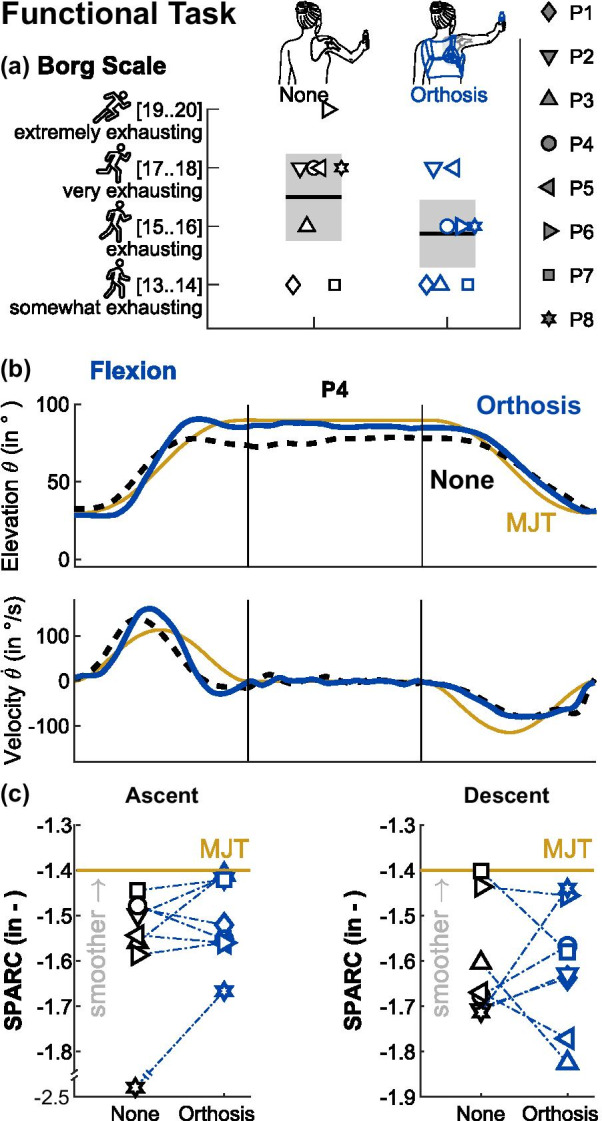
Results for the functional task. Symbols represent participants. **a** With orthosis assistance, participants rated their perceived exertion while elevating a weight 1.25 points lower on the Borg Scale. Black bars represent means, grey boxes represent 95% confident intervals (standard error of the mean). **b** To keep fatigue to a minimum, participants were asked to elevate the weighted bottle in flexion direction only. Exemplary angular position and velocity data for one elevation (ascent—hold weight—descent) from participant P4, overlayed with the respective minimum jerk trajectory (MJT). **c** Movement smoothness in terms of the dimensionless SPARC metric for arm ascent and descent. The MJT represents an upper bound for movement smoothness

With orthosis assistance, participants perceived the elevation of the weighted bottle as less exhausting (average 15.25 points on the Borg Scale) than without assistance (16.5 points), see Fig. 6a.

Performing smooth movements requires less effort than jerky movements with extensive secondary movement components [42, 43]. The ascent and descent sections of the arm elevations were thus evaluated using the SPARC movement smoothness criterion [40]. In general, arm ascent was smoother than arm descent, see the exemplary movement trajectory in Fig. 6b. With the orthosis, three participants came very close to the upper bound of − 1.4, calculated from a minimum jerk trajectory, while three participants moved slightly less smoothly, see Fig. 6c. During arm descent, this effect was less pronounced, with an equal number of participants moving both more and less smoothly with orthosis assistance.

### Subjective ratings of orthosis design

**Fig. 7:**
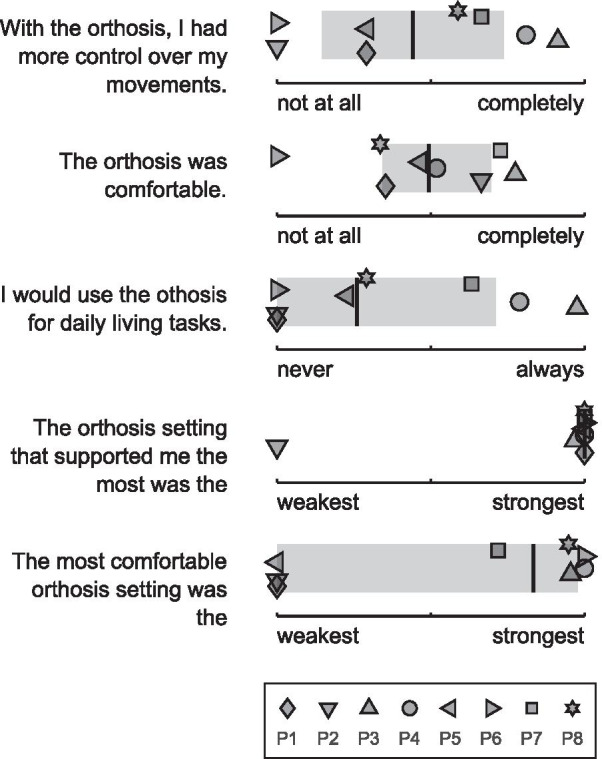
Subjective ratings of orthosis design. For each question, symbols represent participant responses. The vertical black bars represent the median, while the grey shaded boxes represent the 25% and 75% percentiles of data points

At the end of the study, participants were asked a series of questions on their subjective rating of the orthosis design, see Fig. 7. Acceptance of the orthosis varied among participants. While some deemed the orthosis both helpful and comfortable and would consider wearing it for activities of daily living, others did not agree with this rating, or only in part. Except for one, all participants rated the strongest orthosis setting as the most helpful. Four participants furthermore rated the strongest orthosis setting as the most comfortable, while three were of the contrary opinion.

## Discussion

An absent scapular constraint, as in scapular winging, results in substantial loss of arm function, particularly when the arm is elevated in flexion direction [1, 5]. In this study, external scapula assistance—i.e., non-invasive, reversible assistance provided by either a trained therapist (therapist assistance) or a textile-based scapula orthosis (orthosis assistance)—has proven an effective tool to improve shoulder function in humans with an irreversible scapula alata due to muscular dystrophy. Next to improvements in range of motion, perceived exertion could be reduced when the scapula orthosis was applied in a task inspired by activities of daily living.

When receiving external scapula assistance, the majority of participants were able to instantaneously reach higher with reduced perceived exertion. The consistent and instantaneous nature of the improvements seen in this study strongly suggests that external scapula assistance has the potential to effectively increase upper limb function, and is therefore a feasible, more flexible and much less invasive alternative to surgical interventions during therapy and in daily life.

To date, surgical interventions are still the most prevalent method for the treatment of irreversible scapulae alatae, often performed as a treatment for chronic nerve palsies. In combination with subsequent physical therapy, studies on successful surgical interventions reported large range of motion improvements of 30° to 65° in shoulder flexion [21, 25–27], and 25° to 56° in shoulder abduction [20–27]. However, considering the invasiveness of surgical treatment, it has to be noted that failed surgical stabilization attempts are mentioned but rarely reported [44], and controlled clinical trials are unavailable [18]. Though the mean improvements in this study were lower when compared to above surgical interventions, single individuals could benefit from improvements of up to 42.6°/21.2° (therapist/orthosis) in flexion and up to 28.4°/10.7° in abduction when provided with external scapula assistance. These instantaneous gains allow for more physiological movements during activities of daily living. For example, even the average achieved gain of 6.2° with orthosis assistance in flexion roughly complies with the mean difference of 6.5° between pathological and physiological drinking in a study on hemiparetic stroke [45].

Concerning assistance effectiveness, therapist assistance represented an upper bound for ideal external scapula assistance. Indeed, in this study, the therapist outperformed the scapula orthosis in two key aspects: first, the therapist was able to adjust the assistive force as needed throughout the course of movement; second, the therapist was able to actively rotate and, therefore, dynamically stabilize the scapula, bringing the movement pattern closer to the physiological scapulohumeral rhythm. In contrast, the orthosis provided only static assistance, hence dynamic adjustments to a changing situation were not possible. As expected, participants were able to capitalize on the therapist much more than on the orthosis. In abduction direction, when the scapula contributes to arm elevation by rotating upwards laterally, therapist assistance was on average almost twice (factor 1.9) as effective as static orthosis assistance. In flexion, where arm elevation is particularly hampered by the dorsal protrusion of the scapula, therapist assistance was on average almost four times (factor 3.7) as effective. The larger factor in flexion can be explained by the more efficient force application during therapist assistance at the inferior angle of the scapula. Indeed, participants preferred the application of larger forces, as all but one participant deemed the strongest orthosis setting the most helpful. One participant estimated that the orthosis assistance mimics the therapist assistance by 75%, which could be increased with a better fit and larger compression.

In physiological shoulder movement, holding and manipulating weights reduces the upward rotation of the scapula in the lower quadrant of arm elevation, as the muscles around the scapula increase their co-contraction to provide stability [29]. The orthosis applied in this study had an effect comparable to co-contracting muscles in the upper back, constraining the scapula on the thoracic wall. Therefore, it was expected that the orthosis would benefit participants particularly during the manipulation of a weighted object—here in the form of a bottle—similar to the study from Jakab et al. on surgical scapula fixation, in which participants reported subjective postoperative improvements during activities of daily living [26]. Among the participants that picked the lighter bottle (250 g), all but one reported a reduction in perceived exertion with orthosis assistance. The three participants that picked the heavier bottle (500 g) did not report a change in perceived exertion, which they generally rated comparably low. This suggests that the ability of these participants may have surpassed the challenge of the additional weight. Though significant increases in quantitative measures such as movement smoothness and lifting height of the bottle were not observed, the anchoring of the scapula by means of an assistive orthosis improved perceived ability, confidence, and comfort of people with an irreversible scapula alata.

In accordance with the evidence presented in this study, the majority of scapula stabilizing interventions in the literature reported a larger improvement of range of motion in flexion direction when compared to abduction [16, 21, 25, 27]. This finding might be based on the fact that arm elevation is depending much more on the lateral upward rotation of the scapula in abduction, and that most methods provide purely static, i.e., non-rotating support to the scapula. Nonetheless, the current approach in the literature for evaluating scapula stabilizing interventions is to primarily report on shoulder elevation in abduction. Anatomically, an elevation in abduction minimizes strain in the rotator cuff [38] and is therefore intuitively favorable. However, flexion is the most common direction of shoulder elevation in daily life [37] and should therefore be the focus of functional outcomes in the development of assistive devices in the future.

The range of benefit of the current orthosis design was quite wide: While for some participants, orthosis assistance was as effective as therapist assistance, others were obstructed by the device and complained about its poor fit. A strong relationship between participants’ baseline ability, Brooke score or Scapula Alata (ScAla) score and their respective improvement potential was not found. Hence, the effective improvement when therapist assistance is provided, along with the willingness of the person affected by the scapula alata to use an assistive device, constitute the decisive criteria for orthosis suitability. Though the modified Scapula Assistance Test [28], or Horwitz maneuver [27], is a common inclusion criterion for surgical intervention studies on scapula alata treatment, the results of these tests are usually not reported. Often, humans with an irreversible scapula alata acquire individual compensation mechanisms that need to be adapted to each new form of assistance. Hence, long-term adaptation and training may further enhance the effect of orthosis assistance. Additionally, augmenting the scapula orthosis to dynamically assist the rotation of the scapula during arm elevation, similar to therapist assistance, could greatly enhance its usability as an assistive device for daily living activities, as well as the applicability of such a device in rehabilitation of reversible scapulae alatae and in injury prevention at the workplace and during sports activities. Gathering more data in this direction may help to improve usability and suitability criteria for scapula orthoses in the future.

Gross compensatory trunk movements were observed during arm elevations, though participants were instructed to maintain an upright posture while seated on a stool without backrest. Facilitating trunk movements are common even in physiological reaching [3]. However, to compensate the lack of scapula rotation, participants adopted excessive trunk movements in the plane of arm elevation to increase their workspace. When assisted by the orthosis, the majority of participants maintained or reduced their compensatory trunk movements, aligning their movement pattern closer to physiological reaching [3]. In combination with testimonial evidence, we conclude that the orthosis compression enabled a more upright posture during arm elevation and a more compact, integrated feeling of the upper limb. A similar effect was seen for therapist assistance, though the human–human interaction made it impossible to isolate the effect of external scapula assistance on compensatory trunk movements in this case.

Current advances in robotic technologies, such as the emergence of soft exosuits, are raising the hope for textile-based assistive technologies that complement therapists in daily life scenarios. This may boost training intensity, rehabilitation efficacy and patient independence. Exosuits can assist the upper extremity against gravity during arm elevation [46–48]. However, when the stability of the scapula is compromised, unphysiological movement patterns can occur that may lead to further joint injury. Especially for muscular dystrophy patients, a full recovery from an injury is uncertain, and therefore to be avoided. To date, upper limb exosuits with scapula assistance do not exist. The scapula orthosis presented in this study represents a starting point to increase the accessibility of this technology for people with scapular dyskinesis, for example due to a scapula alata.

Though scapula assistance is a basic element of physical therapy, e.g., after stroke or shoulder injury, it is exclusively limited to direct therapist–patient treatments. Just for unilateral treatment, the therapist must guide the scapula in addition to the upper and forearm and stabilize the trunk. By using a wearable scapula orthosis in clinical practice, the involvement of the therapist can be transformed ergonomically to pure arm support. To further relieve the therapist of the physical burden, therapy robots have been developed. Taking the form of exoskeletons, these robots guide and support the arm against gravity [46, 49]. While exoskeletons effectively complement the treatment of more able-bodied patients, patients with a scapula alata cannot benefit from the full capacity of the robot, since the typical lack of a shoulder support mechanism can lead to further injuries. Complementing physical therapy with a wearable scapula orthosis, comparable to the one presented in this study, may enhance patient safety, physical and occupational therapy options and applicability of therapy robots.

## Conclusion

External scapula assistance is an effective tool for providing instantaneous support to the upper limb of people with scapular dyskinesis due to a scapula alata. In this study, therapist assistance proved to be more effective than orthosis assistance, showing the potential for further improvements of this technology. Already in its current form, the scapula orthosis is a feasible tool for scapula assistance. With a further refinement of the design based on future longitudinal and usability focused studies, the scapula orthosis and its scope of application can be enhanced for augmenting therapy and rehabilitation technology, for enabling daily life assistance, and, consequently, for improving the overall quality of life of people with scapula alata.

## Supplementary Information

**Additional file 1.** Orthosis Sewing Patterns.

**Additional file 2.** Angle Definition Visualizations.

**Additional file 3.** Motion Capture Error Analysis.

**Additional file 4.** Plane of Elevation Analysis.

**Additional file 5.** Supplementary Results.

**Additional file 6.** Movie file "Reaching higher: External scapula assistance can improve upper limb function in people with irreversible scapula alata.

## Data Availability

The data generated and analyzed during this study are available in the online repository at https://doi.org/10.5281/zenodo.4436142 and may be re-used for ethical, scientific purposes.

## Abbreviations

ScAla: Scapula Alata Score
FSHD: Facio-scapulo-humeral dystrophy
MYOS: Necrotizing myositis
CALP: Calpainopathy
*CI*_95%_: 95% Confidence interval in terms of the standard error of the mean
