# Supplementary material for: Reaching higher: External scapula assistance can improve upper limb function in humans with irreversible scapula alata
Source: J Neuroeng Rehabil. 2021 Sep 3;18:131. doi: 10.1186/s12984-021-00926-z (PMC8414749; doi:10.1186/s12984-021-00926-z)
Supplement: Supplementary file 2 — Additional file 2. Angle Definition Visualizations. [file 12984_2021_926_MOESM2_ESM.pdf]

Angle Definition Visualizations

Arm elevation angle with respect to gravity θ_g

- defined locally in the plane of elevation as the angle between the wrist vector $\vec{\mathbf{w}}$, connecting the shoulder and the wrist, and the gravity vector $\vec{\mathbf{g}}$ during peak arm elevation.

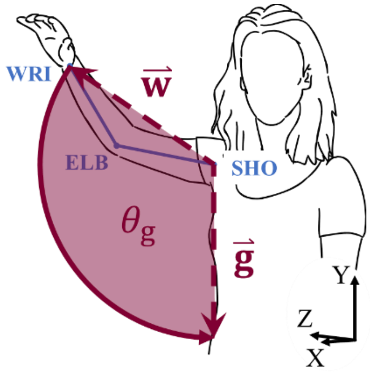

Trunk compensation angle κ

- defined as the differential of the angle between the sternum vector $\vec{\mathbf{s}}$ and the gravity vector $\vec{\mathbf{g}}$ between current arm elevation and the initial position, projected onto the plane of elevation.

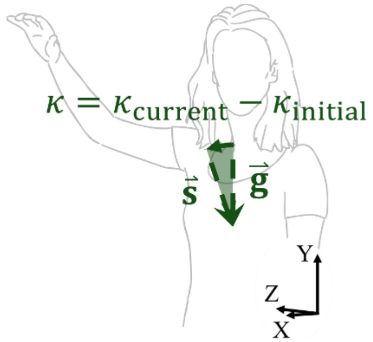

Arm elevation angle θ

- defined as $\theta = \theta_g - \kappa$ that is the gravitational elevation angle θ_g corrected for the thorax compensation angle κ.

Plane of elevation

- defined as the plane spanned by the wrist vector and the gravity vector.

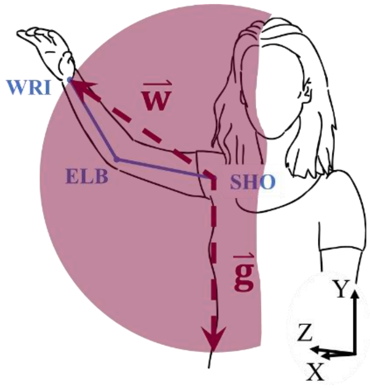

Plane of elevation angle α

- defined as the angle between the projections of the line connecting both acromia in the initial position and the line connecting the glenoid with the wrist onto the horizontal plane. Flexion was defined in correspondence with the primary direction during arm elevation occurring in daily life at $\alpha = 80°$ plane of elevation angle [30]. Abduction was defined in correspondence to the scapular plane at $\alpha = 30°$ plane of elevation angle

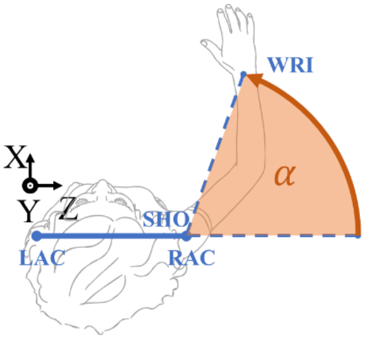

top view

Scapula rotation angle ρ

- defined as the angle between the medial border of the scapula and the gravity vector.

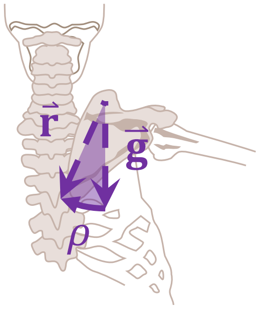

rear view

Glenohumeral elevation angle θ_{GH}

- defined as $\theta_{GH} = \theta_g - \rho$, which corresponds to the angle between the humerus and the scapula rotation angle ρ.

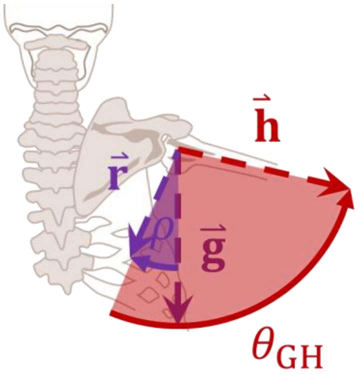

rear view
