# Supplementary material for: Reaching higher: External scapula assistance can improve upper limb function in humans with irreversible scapula alata
Source: J Neuroeng Rehabil. 2021 Sep 3;18:131. doi: 10.1186/s12984-021-00926-z (PMC8414749; doi:10.1186/s12984-021-00926-z)
Supplement: Supplementary file 3 — Additional file 3. Motion Capture Error Analysis. [file 12984_2021_926_MOESM3_ESM.pdf]

Motion Capture Error Analysis

The maximum error in the elevation angle analysis due to soft tissue displacement and motion capture data processing typically remained below 1°, see **Error! Reference source not found.**.

Model for maximum error estimation

$$\sin\left(\frac{\hat{\theta}_e}{2}\right) = \frac{\frac{1}{2}\cdot 1.96\cdot \sigma_{d_w}}{\ell_w - 1.96\cdot \sigma_{\ell_w}}$$

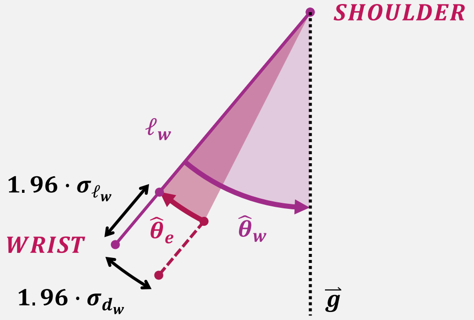

With maximum angle error θ_e, standard deviation of wrist width σ_{d_w}, standard deviation of humeral length σ_{ℓ_w}, and mean humeral length ℓ_w.

Approximation for small angles via arc length:

$$1.96\cdot\sigma_{d_w} = \frac{\hat{\theta}_e}{2\pi}\cdot\left(2\pi\left(\ell_w - 1.96\cdot\sigma_{\ell_w}\right)\right)$$

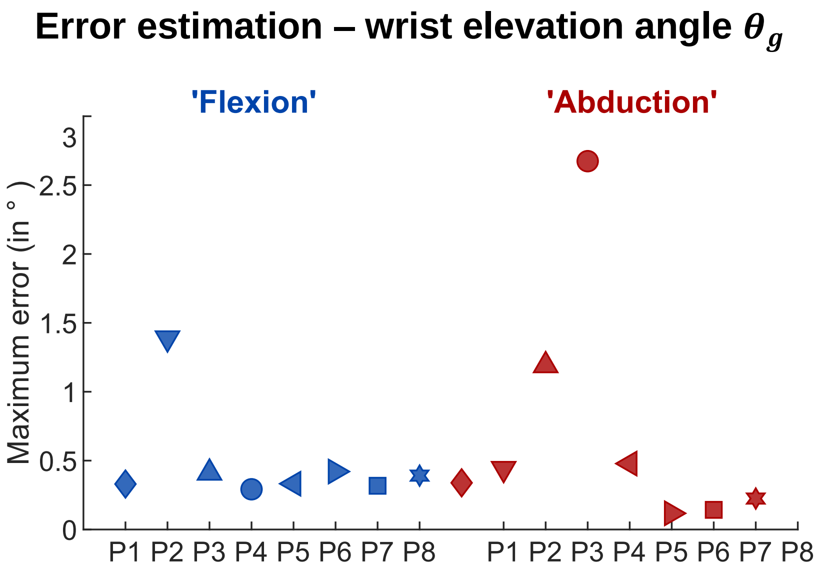

Estimation of maximum errors in wrist elevation angle calculation due to marker errors (marker reconstruction and soft tissue displacement, see ***Error! Reference source not found.*** *and* ***Error! Reference source not found.****).*

Motion capture errors – data processing

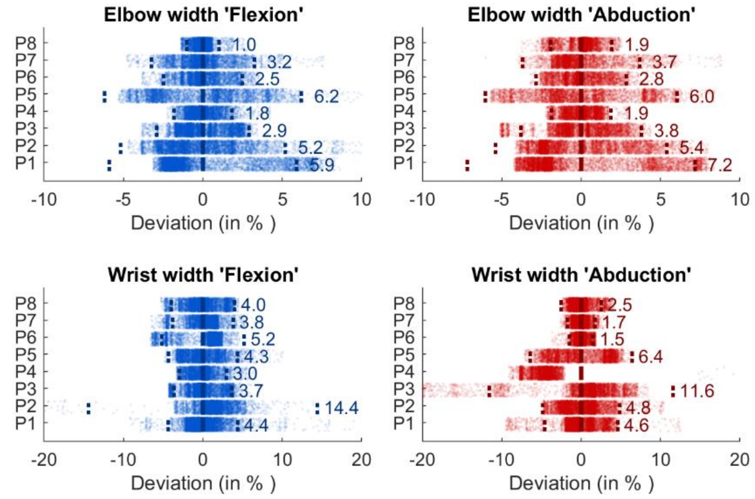

Motion capture errors due to data processing. Errors in wrist and elbow width (here, markers were placed on bony landmarks) correlate with residual errors in the motion capture data processing and errors due to marker reconstruction.

Motion capture errors – tissue displacement

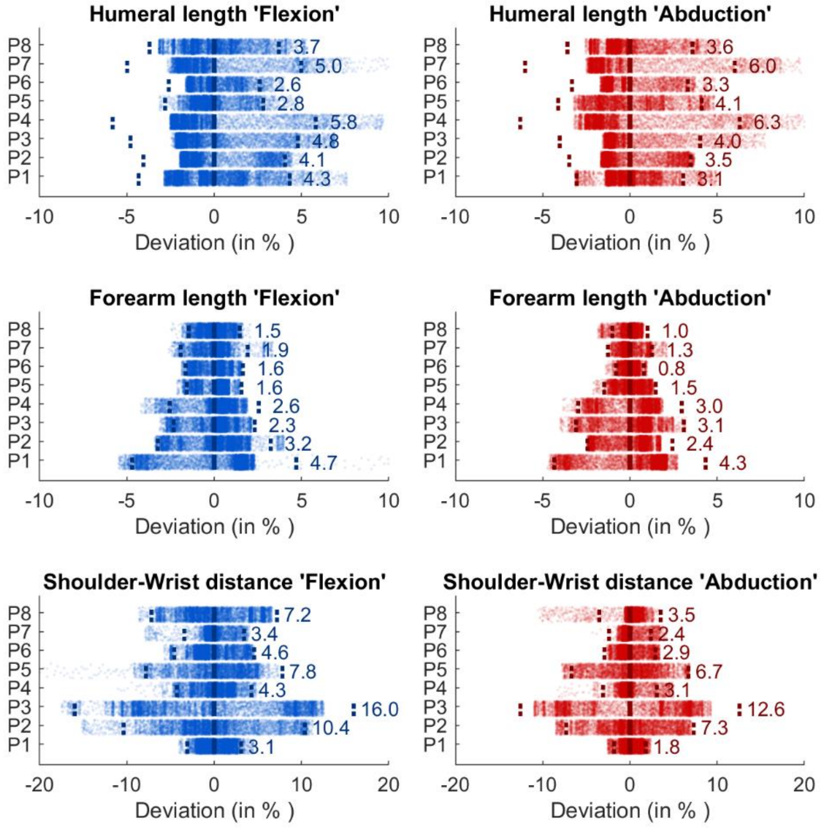

Motion capture errors due to soft tissue displacement. Especially at the shoulder during arm elevation, soft tissue displacement correlates with changes in arm length, in particular the humeral length and shoulder-wrist distance.
