# Supplementary material for: Reaching higher: External scapula assistance can improve upper limb function in humans with irreversible scapula alata
Source: J Neuroeng Rehabil. 2021 Sep 3;18:131. doi: 10.1186/s12984-021-00926-z (PMC8414749; doi:10.1186/s12984-021-00926-z)
Supplement: Supplementary file 4 — Additional file 4. Plane of Elevation Analysis. [file 12984_2021_926_MOESM4_ESM.pdf]

Plane of Elevation Analysis

This analysis serves to investigate how participants fulfilled the instruction “elevate your arm towards the target point”. The underlying assumption is that participants aimed at the target with their hand/fingertips. Therefore, the wrist plane of elevation rotation should coincide with the target angle. However, if the arm is not fully extended, the humeral plane of elevation does not necessarily coincide with the target angle. The reference line for the coordinate frame (Z-axis) is the (average) line connecting the left with the right acromion, pointing towards the right acromion.

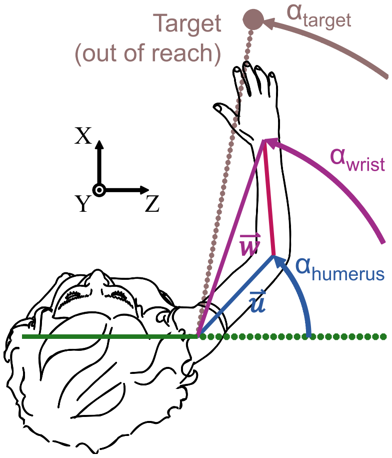

$$\vec{ac} = \overline{RAC} - \overline{LAC}$$

The plane of elevation is the plane spanned by the vector $\vec{u}$ connecting the shoulder with the elbow (humeral PoE) or the vector $\vec{w}$ connecting the shoulder and the wrist (wrist PoE), respectively. The plane of elevation rotation α is the angle between the PoE and the Z-axis.

$$\vec{u} = \overline{ELB} - \overline{SHO}$$

$$\alpha_{humerus} = \cos^{-1}\left(\frac{\vec{ac} \circ \vec{u}}{|\vec{ac}| \cdot |\vec{u}|}\right)$$

$$\vec{w} = \overline{WRI} - \overline{SHO}$$

$$\alpha_{wrist} = \cos^{-1}\left(\frac{\vec{ac} \circ \vec{w}}{|\vec{ac}| \cdot |\vec{w}|}\right)$$

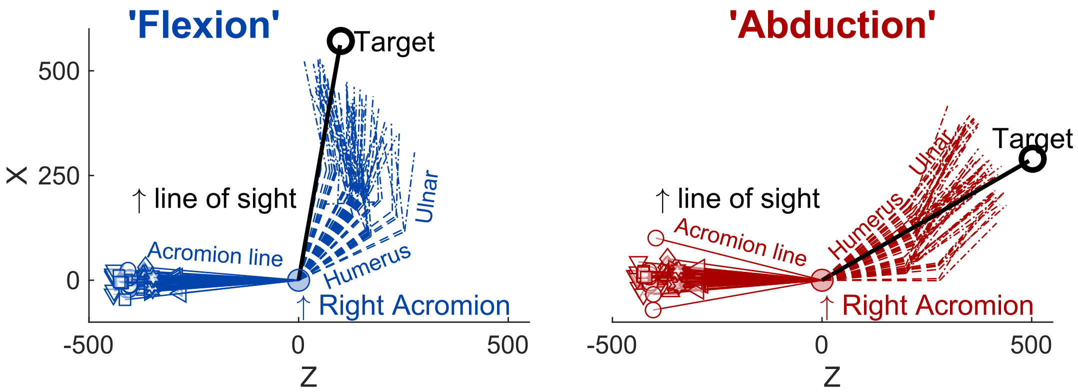

Birds eye view of maximal arm elevations.

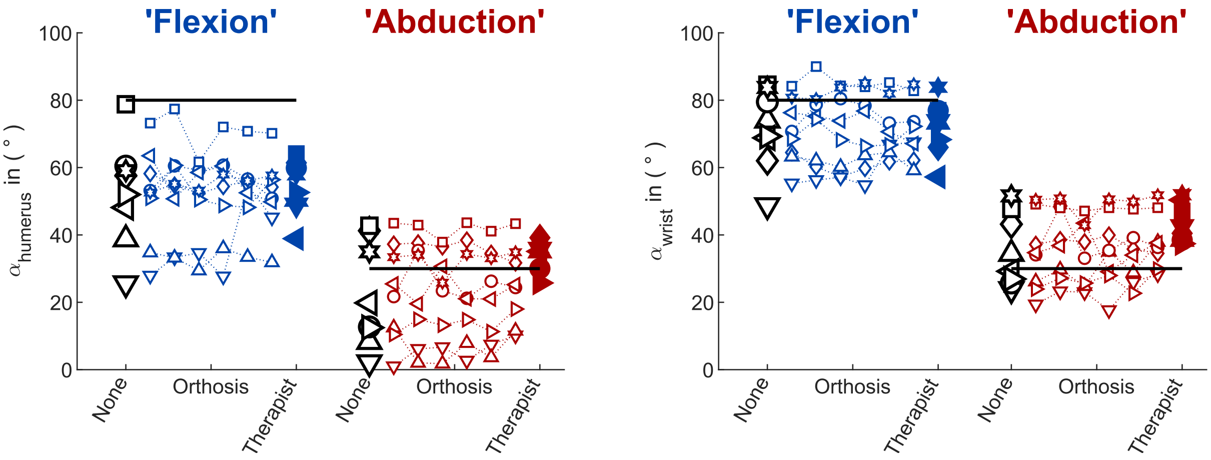

Plane of elevation Analysis. When analyzing the direction of arm elevation (plane of elevation), it becomes apparent that participants were aiming for the target pole with their hand (α_{wrist}) rather than their upper arm ($\alpha_{humerus}$). Therefore, in this study, arm elevation data was analyzed in terms of the line connecting the shoulder and the wrist.
