# Supplementary material for: Reaching higher: External scapula assistance can improve upper limb function in humans with irreversible scapula alata
Source: J Neuroeng Rehabil. 2021 Sep 3;18:131. doi: 10.1186/s12984-021-00926-z (PMC8414749; doi:10.1186/s12984-021-00926-z)
Supplement: Supplementary file 5 — Additional file 5. Supplementary Results. [file 12984_2021_926_MOESM5_ESM.pdf]

Range of motion task: Borg scale ratings

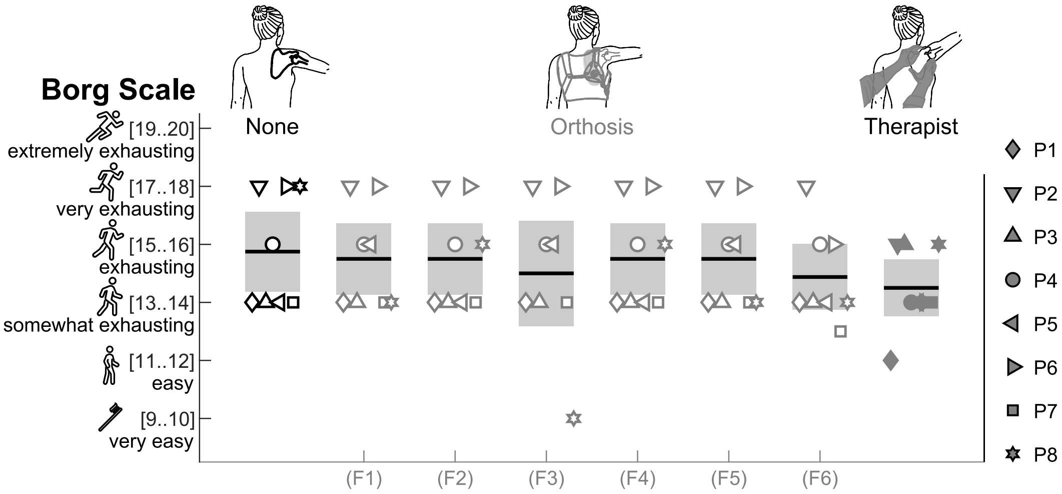

Borg Scale results for perceived exhaustion during the range of motion task. In accordance with range of motion and qualitative results, participants perceived the strongest (F6) as the least exhausting orthosis setting, and the therapist assistance as the least exhausting overall. Symbols represents participants, black bars represent medians, grey boxes represent 25-/75-percentiles of data.

Functional task: Elevation and Trunk compensation

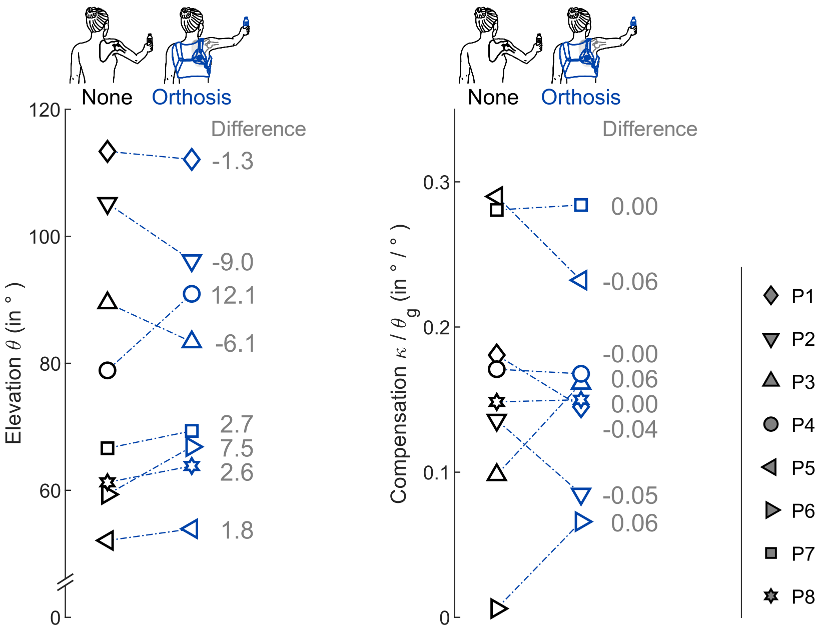

Range of motion in terms of bottle lifting height (left) and trunk compensation normalized by lifting height w.r.t. gravity (right) for the functional task. In particular participants with lower baseline ability were able to improve their lifting height with the orthosis, while participants with better baseline ability seemed to be restricted. The effect of the scapula orthosis on trunk compensation was inconclusive, with the same number of participants increasing as reducing their compensation.
