# Supplementary material for: Reaching higher: External scapula assistance can improve upper limb function in humans with irreversible scapula alata
Source: J Neuroeng Rehabil. 2021 Sep 3;18:131. doi: 10.1186/s12984-021-00926-z (PMC8414749; doi:10.1186/s12984-021-00926-z)

Reaching higher: External scapula assistance improves upper limb function in humans with irreversible scapula alata

Scapula Orthosis - Sewing patterns

Authors: Anna-Maria Georgarakis[1,2]*, Michele Xiloyannis[1,2], Christian Dettmers[3], Michael Joebges[3], Peter Wolf[1], Robert Riener[1,2]

Affiliations:

[1]Sensory-Motor Systems (SMS) Lab, Institute of Robotics and Intelligent Systems (IRIS), Department of Health Sciences and Technology (D-HEST), ETH Zurich, Switzerland

[2]Reharobotics Group, Spinal Cord Injury Center, Balgrist University Hospital, Medical Faculty, University of Zurich, Switzerland

[3]Kliniken Schmieder Konstanz, Konstanz, Germany

***marie.georgarakis@outlook.com**

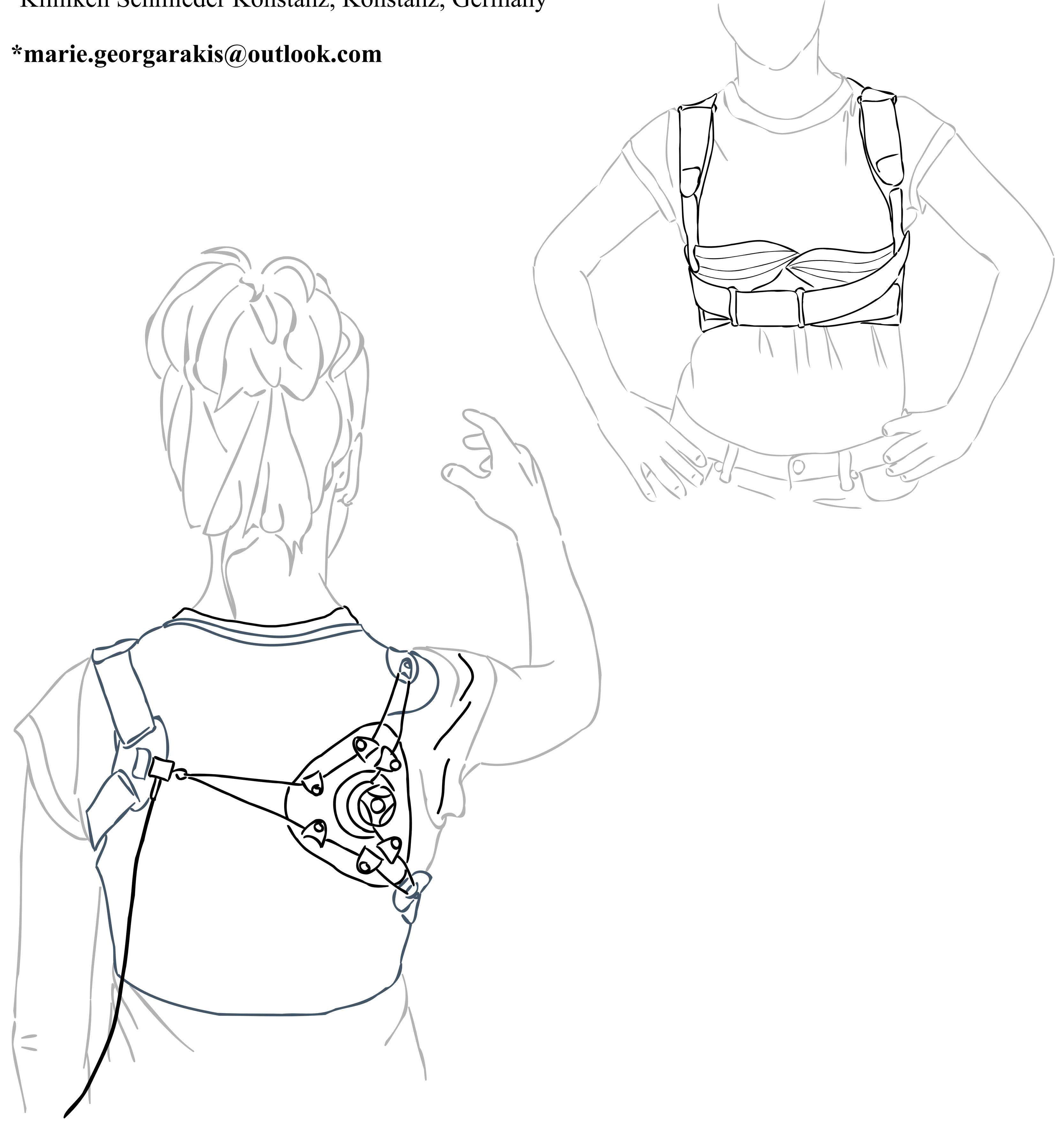

Tayloring components / elements

BOA pressure plate

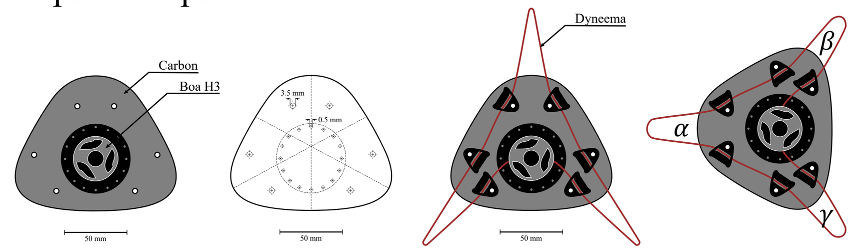

Triglide buckles and D-Rings

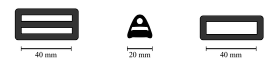

Croco-Velcro

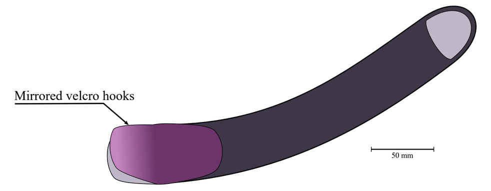

Buckle straps

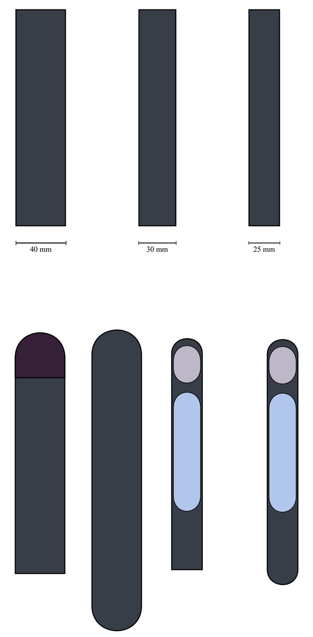

Materials

- Cordura 1000den, Invista, Switzerland
- Cordura 500den, Invista, Switzerland
- Cordura 500den, Invista, Switzerland
- Synthetic leather
- Seatbelt webbing
- Velcro hook patches
- Velcro loop patches

outside

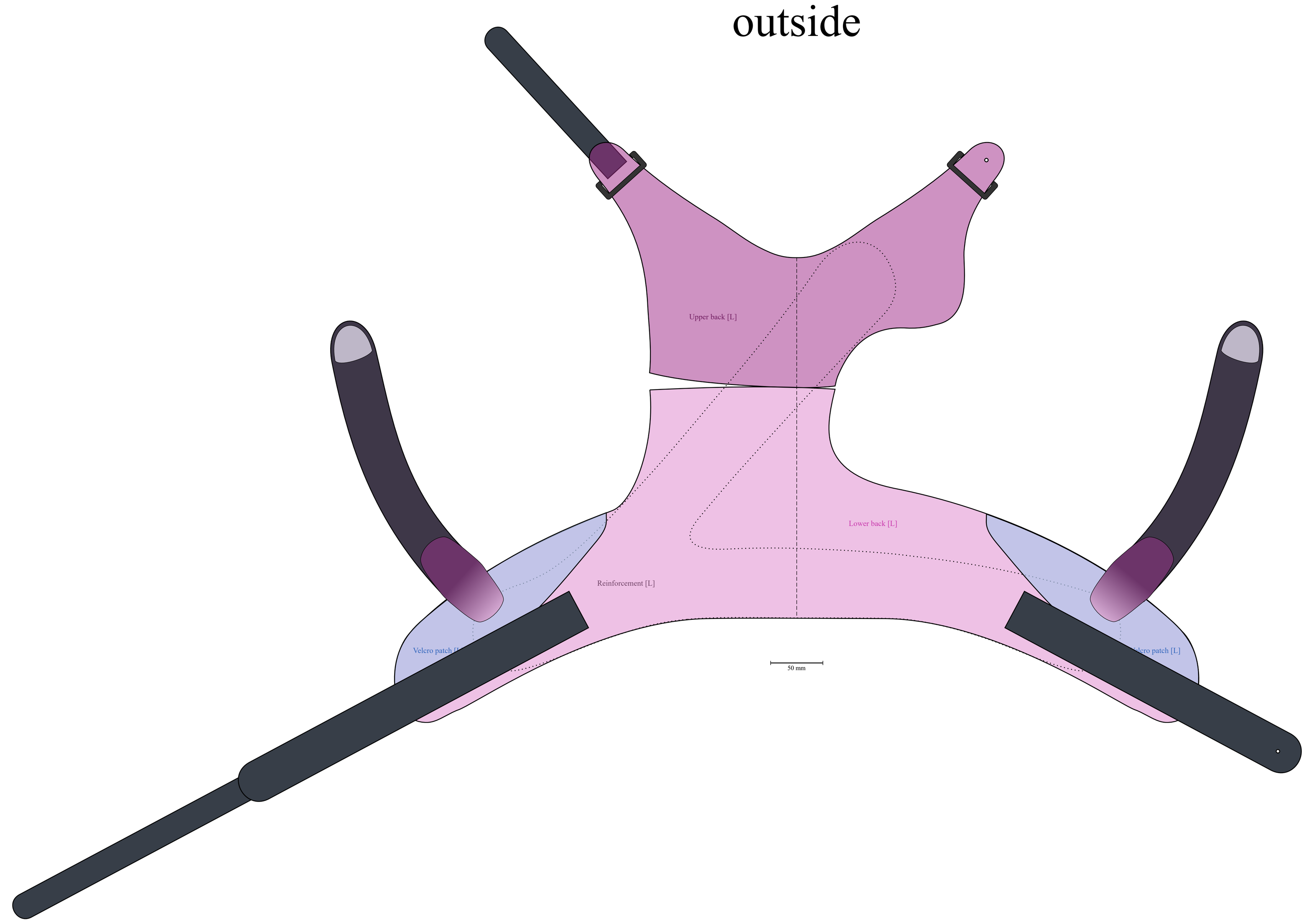

inside

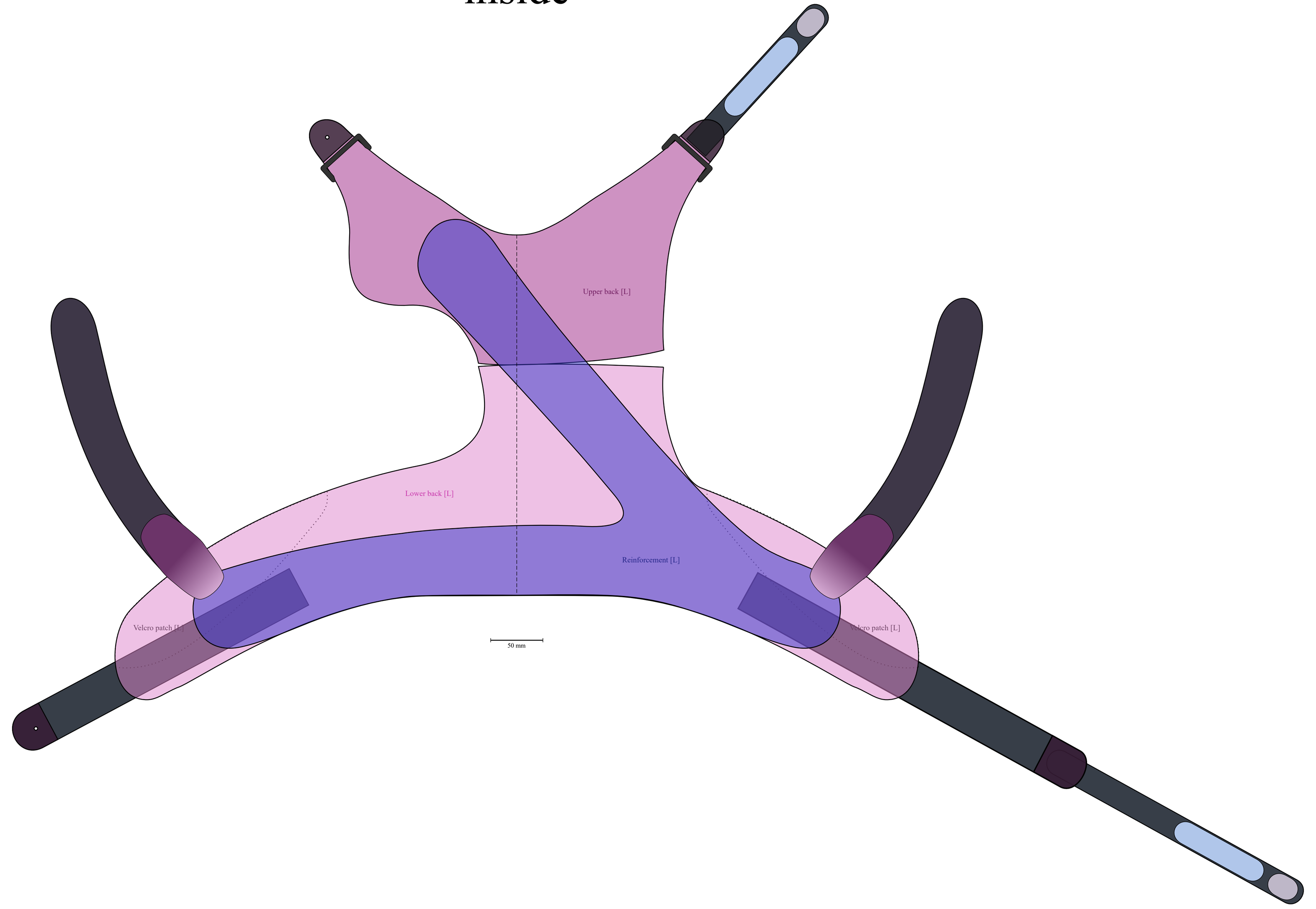

Orthosis harness [L]

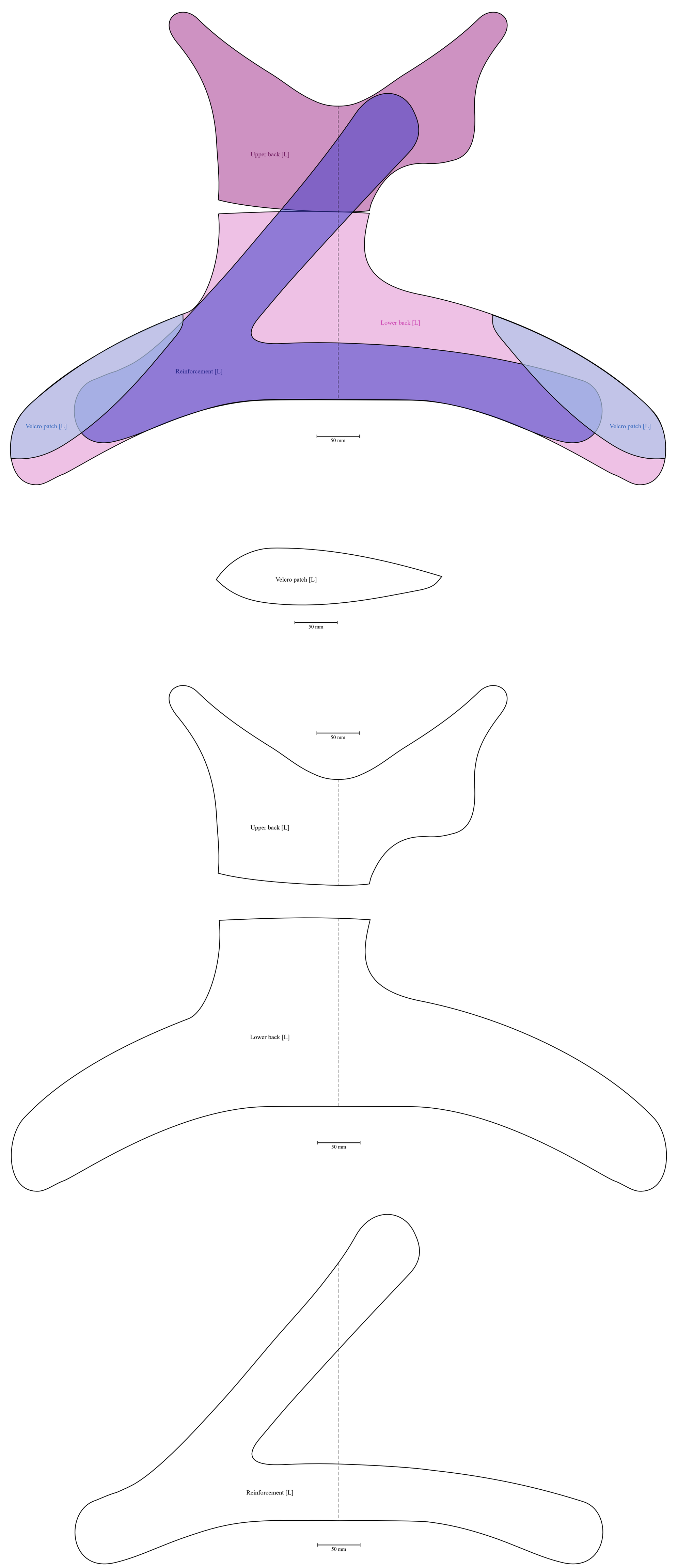

Orthosis harness [S]

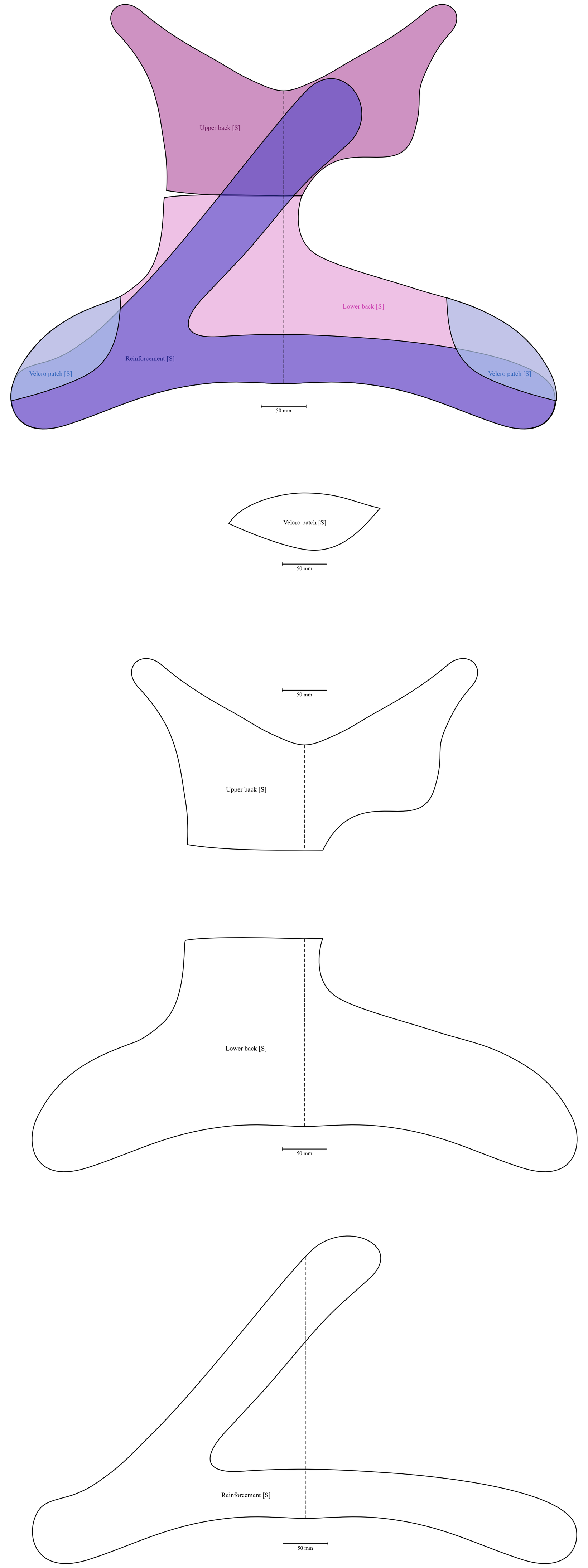

Supplement: Supplementary file 1 — Additional file 1. Orthosis Sewing Patterns. [file 12984_2021_926_MOESM1_ESM.pdf]
